# MRI-Based Radiomics Analysis for Intraoperative Risk Assessment in Gravid Patients at High Risk with Placenta Accreta Spectrum

**DOI:** 10.3390/diagnostics12020485

**Published:** 2022-02-14

**Authors:** Caiting Chu, Ming Liu, Yuzhen Zhang, Shuhui Zhao, Yaqiong Ge, Wenhua Li, Chengjin Gao

**Affiliations:** 1 Xinhua Hospital Affiliated to Shanghai Jiao Tong University School of Medicine, Shanghai 200092, China; chucaiting@xinhuamed.com.cn (C.C.); liuming01@xinhuamed.com.cn (M.L.); zhangyuzhen@xinhuamed.com.cn (Y.Z.); zhaoshuhui@xinhuamed.com.cn (S.Z.); 2 GE Healthcare, Pudong New Town, No. 1, Huatuo Road, Shanghai 201203, China; Yaqiong.Ge@ge.com

**Keywords:** radiomics, MRI, placenta accreta spectrum, high risk

## Abstract

Background: Gravid patients at high risk with placenta accreta spectrum (PAS) face life-threatening risk at delivery. Intraoperative risk assessment for patients is currently insufficient. We aimed to develop an assessment system of intraoperative risks through MRI-based radiomics. Methods: A total of 131 patients enrolled were randomly grouped according to a ratio of 7:3. Clinical data were analyzed retrospectively. Radiomic features were extracted from sagittal Fast Imaging Employing State-sate Acquisition images. Univariate and multivariate regression analyses were performed to build models using R software. A receiver operating characteristic curve and decision curve analysis (DCA) were performed to determine the predictive performance of models. Results: Six radiomic features and two clinical variables were used to construct the combined model for selection of removal protocols of the placenta, with an area under the curve (AUC) of 0.90 and 0.91 in the training and test cohorts, respectively. Nine radiomic features and two clinical variables were obtained to establish the combined model for prediction of intraoperative blood loss, with an AUC of 0.90 and 0.88 in the both cohorts, respectively. The DCA confirmed the clinical utility of the combined model. Conclusion: The analysis of combined MRI-based radiomics with clinics could be clinically beneficial for patients.

## 1. Introduction

Placenta accreta spectrum (PAS) represents a heterogeneous group of abnormal placental implantation, such as placenta accreta, placenta increta, and placenta percreta, based on the different depths of villi invasion from the myometrium to the uterine serosa [1]. PAS incidence increases annually and is estimated to be over 9000 per year by 2020 [2]. Among them, a history of previous cesarean section (CS) and placenta previa are strongly associated with the prevalence and incidence of PAS [3,4]. Herein, pregnant women with a history of prior CS or present placenta previa are viewed as gravid patients at high risk with PAS [5,6]. Such patients face serious risks after delivery of the fetus, such as placental residue, life-threatening hemorrhage, and even death, which are closely related to the breadth and depth of abnormal placental implantation [3,7]. Hysterectomy is recommended as a safe management plan for patients with PAS, owing to the effective control of major hemorrhage. The corresponding deficiency is the loss of fertility of patients [8]. Therefore, patients who want to preserve their fertility must be willing to choose conservative managements in cases of safety to be guaranteed [9]. The method to develop an appropriate treatment plan for these patients, including conservative surgical treatment along with hemorrhage control, is yet to be solved.

At present, Ultrasound (US) as the first-line examination and magnetic resonance imaging (MRI) are the two prenatal mainstay diagnostic methods, which are less involved in clinical risk management or guidance treatment [10,11,12,13,14]. Hence, effectively used imaging for prenatal evaluation of intraoperative risk to guide the treatment for patients is anticipated for clinics. Radiomics, which refers to the high-throughput extraction of a large number of imaging features from medical images to assist accurate diagnosis, has become popular in clinical research [15]. Advantages include not only avoiding subjective judgement but also incorporating the complete use of objective information. Predictive models or nomograms, which are developed by radiomics signature as well as clinical data, could offer intuitive imaging biomarkers for diagnosis, guide management, and assessment of prognosis [16,17]. Available articles on placental radiomics are mainly concerned with diagnosis, assessment of postpartum hemorrhage and prediction of hysterectomy for patients with PAS; however, articles on intraoperative risk assessment are few [18,19,20,21].

In this study, we aimed to establish MRI-based radiomic features in gravid patients at high risk for PAS, and develop an assessment system of intraoperative risk including the schemes of the placental stripping and assessment of accompanying bleeding for achieving comprehensive evaluation and individualized treatment, which was superior over a single prediction for hysterectomy or postpartum hemorrhage in the current articles. Fortunately, the abstract of this study has been accepted as a poster exhibition of the upcoming RSNA 2021 Annual Meeting.

## 2. Results

### 2.1. Patient Characteristics

Of the 131 patients with CS, 73 patients underwent active separation of the placenta, and 58 patients received manual stripping of the placenta, six of whom had to further undergo hysterectomy. Unexpectedly, 6 of 63 patients at high risk with non-PAS receive manual stripping of the placenta. Meanwhile, there were 95 patients with IBL of less than 1000 mL and 36 patients with IBL greater than or equal to 1000 mL. In the group with IBL greater than or equal to 1000 mL, there was actually one high-risk patient with non-PAS.

Patient characteristics and their associations with RPP are displayed in Table 1. Of six clinical variables, four clinical variables including weeks of gestation at the time of MRI examination, placenta previa, and number of previous CS as well as previous surgical abortions showed significant difference between the different removal placenta groups in the training cohort. Differences in clinical outcomes such as blood loss during surgery and subtypes of PAS between the two groups with different removal protocols were statistically significant in the whole cohort. 

Table 2 shows all the patient characteristics and their association with IBL. Similarly, four of the six clinical variables had significant differences between groups with IBL of less than 1000 mL and of greater than or equal to 1000 mL in the training cohort, which were as followings: weeks of gestation at the time of MRI examination, previa placenta, and number of previous CS or surgical abortions. Significantly statistical differences in clinical outcomes including RPP and subtypes of PAS were also observed between the two groups with different bleeding volumes in the whole cohort. 

### 2.2. Features Selection and Development and Validation of Prediction Models

Results for the selection of RPP are shown below in detail. After the univariate analysis, four clinical variables with statistical differences including weeks of gestation at the time of MRI examination, placenta previa, and number of previous CS as well as surgical abortion were obtained and then used to develop a clinical predictive model by univariate regression analyses. Of all the 1130 radiomics features, 6 key features were selected by mRMR algorithm and LASSO regression, which were as following: original_glszm_SmallAreaLowGrayLevelEmphasis, wavelet-HLH_glszm_LowGray-LevelZoneEmphasis, wavelet-HHH_glrlm_LongRunHighGrayLevelEmphasis, wavelet-LLL_firstorder_TotalEnergy, wavelet-HLH_firstorder_Kurtosis, and log-sigma-2-0-mm-3D_glcm_Correlation (Supplementary Figure S1), and were furtherly used to build the radiomics predictive model. Subsequently, the Rad score of each patient was calculated using the above six radiomic features as well as related weight weighted coefficients. Finally, the clinical-radiomics combined predictive model and nomogram were developed using the two critical clinical variables and Rad score.

Results section for prediction of IBL were depicted by the following. Likewise, four clinical variables with statistical differences such as the weeks of gestation at the time of MRI examination, placenta previa, and numbers of previous CS as well as surgical abortion were selected and further used to construct the clinical predictive model. Of 1130 radiomic features, 9 critical features including wavelet-HLL_firstorder_RootMean_Squared, wavelet−HLH_glszm_SmallAreaLowGrayLevelEmphasis, wavelet-LLL_firstorder_TotalEnergy, log−sigma−2−0−mm−3D_firstorder_Mean, wavelet−HHL_firstorder_Skewness, log−sigma−2−0-mm−3D_glcm_Correlation, wavelet−LHL_glcm_ClusterPromine, wavelet-HLL_glcm_lmc1, and original_shape_Maximum2DDiameter-Column (Supplementary Figure S1) were obtained. The above nine radiomic features were used to develop the radiomics predictive model and calculate the Rad score of patients. Similarly, the two critical clinical variables and Rad score were used to build the clinical-radiomics combined predictive model and nomogram.

### 2.3. Predictive Performance of All the Models and Clinical Utility

**Figure 1:**
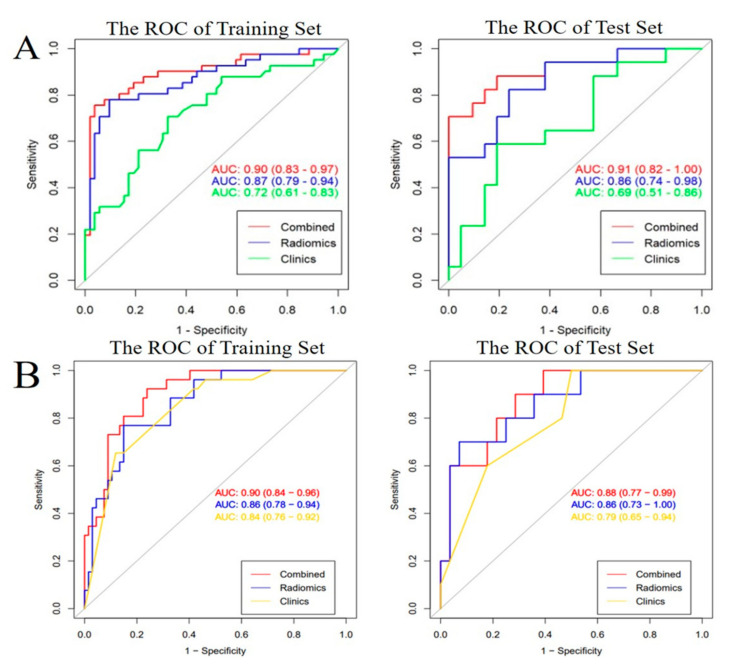
The ROC curves of the radiomics model (blue line), clinical model (green line and yellow line), and clinical-radiomics model (red line) for predicting removal protocols of the placenta (**A**) and intraoperative blood loss (**B**). The predictive performance of clinical-radiomics combined model all outperformed ones of radiomics model or clinical model in both the training and test cohorts.

The performances of the six models, including the clinical model, radiomics model, and clinical-radiomics combined models in the training and test samples are shown in Table 3. Among the models, the combined models for selection of RPP and prediction of IBL were optimal, according to the AUC value (Figure 1).

**Figure 2:**
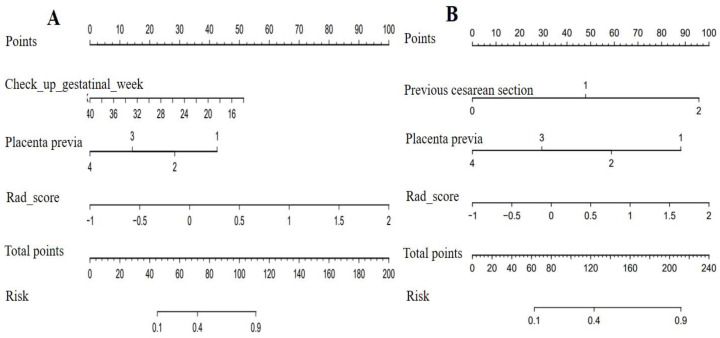
The clinical-radiomics nomogram for predicting removal protocols of the placenta (**A**) and intraoperative blood loss (**B**) was constructed by the Rad score and critical clinical variables. Placenta previa: 4–Low lying; 3–Marginal; 2–Partial; 1–Complete.

**Figure 3:**
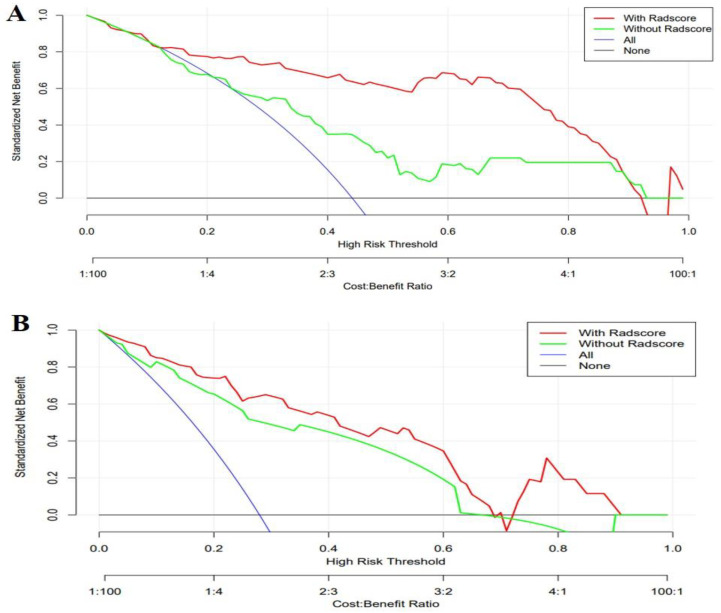
Decision curve analysis for the clinical model and combined model. The decision curve showed that a combined model to predict removal protocols of the placenta at the threshold probability of 12–88% (**A**) and predict intraoperative blood loss at threshold probability of 2–69% as well as 74–92%(**B**) would be more beneficial than the clinical model.

Clinical-radiomics nomograms were built with the selected clinical and radiomic features (Figure 2). The DCA revealed that if the threshold probability was 12–88% for the selection of RPP and 2–69% as well as 74–92% for the prediction of IBL, using the clinical-radiomics combined prediction model would more beneficial than using the clinical model (Figure 3).

## 3. Discussion

Our results indicated that merely making a diagnosis for patients at high risk with PAS does not adequately guide clinical treatment, and clinical-radiomics combined models had a better predictive performance for selection of RPP and prediction of IBL in gravid patients at high risk with PAS, with an improved AUC and a relatively high sensitivity of model as well as specificity of model in the training and validation cohorts, in contrast to the radiomics or clinical models. According to the clinical-radiomics nomogram as an individual and visualized tool, we could evaluate the intraoperative risk to develop a personal surgery scheme for gravid patients at high risk with PAS. Furthermore, a decision curve analysis was used to confirm the clinical benefit. 

### 3.1. The Status and Related Research of Intraoperative Risk Assessment for Patient

To date, there are few data available to directly inform the optimal treatment protocols for patient at high risk with PAS. Obstetricians develop the treatment plans for such patients only with the aid of comprehensive assessment, of which, imaging diagnostic reports such as US and MRI play an important role in it. However, they are highly depending on skill-levels of ultrasound doctors and radiologists’ experiences [2,22]. The rise of radiomics offers a possible way for comprehensive and objective analysis of placental diseases [18,19,23]. It has been well documented that the radiomics features can be objectively predictive for patients with PAS requiring caesarean hysterectomy, with an AUC of 0.80, which was lower than the AUC of 0.86~0.87 for selecting RPP in the study [18]. Regrettably, caesarean hysterectomy remains suboptimal for patients wishing to conserve the uterus. Hence, conservative managements, including extirpative treatment, expectant management, 1-step conservative surgery, and the triple-P procedure, are alternative methods to widely adopt by obstetricians and gynecologists in clinical work [9]. In the study, the majority of patients received active or manual removal treatment of the placenta avoiding a peripartum hysterectomy. Then we have successfully established clinical and radiomics combined model for selection of RPP, with excellent predictive performance. Meanwhile we cannot ignore unmanageable bleeding accompanied with conservative treatment. We thus continued assessment of the second aspect, namely, prediction intraoperative bleeding. Similarly, we constructed the combined model for prediction of IBL. An AUC of 0.9 for prediction of IBL in the study was slightly higher than that of 0.89 for the prediction of postpartum hemorrhage [19]. Although conventional MRI of the placenta contributes to assessment of peripartum complications for patients with PAS such as bleeding, and it mainly focused on the features of T2 black blood sequences, which showed an AUC of 0.80 [14]. In the current study, we adopted bright-blood sequences of T2, which were not applicable for conventional assessment, while we obtained a higher AUC of 0.88~0.9 with help of T2-based radiomics analysis. Thus, our study achieved overall evaluation of intraoperative risk for gravid patients at high risk with PAS.

### 3.2. The Feasibility of MRI-Based Radiomics Analysis for Intraoperative Risk Assessment

Radiomics, making full use of imaging information, can objectively as well as thoroughly reveal the true condition of the placenta so as to have better clinical application prospects, in contrast to traditional imaging [18,24]. The severity and complexity of patients with PAS require rigorous evaluation. Here, the segmented VOI precision of radiomics is critical in follow-up analyses. In this study, we selected sagittal FIESTA images for analysis referring to the relevant literature [19,20,21]. We then identified the boundary of the VOI and segmented VOI combination of conventional image analysis and consensus of inter-observers. However, VOI segmentation repeatedly performed by different radiologists or a radiologist in different time periods would have been more useful to reduce bias in the assessment of derived radiomics features. Considering that the suspicious lesion regions of the placenta had an ill-defined boundary, which was different from the well-defined boundary of tumors, two experienced obstetric radiologists in the study firstly co-analyzed conventional images to identify the suspicious lesion regions, and then determined the maximum margin of lesions including the placenta and uterus, which ensured not only inclusion the suspicious lesion regions but also no omissive lesion. Finally, the third, more experienced obstetric radiologist performed VOI segmentation according to the previous consensus, which was equivalent to repeat analysis and again guaranteed the precision of VOI segmentation. Furthermore, we extracted certain features with the aid of the mRMR algorithm and the LASSO regression, which ensured that the obtained features were optimal [25]. We selected the majority of features belonging to the first-order features and texture features, such as GLSZM and GLCM, which showed spatial distribution patterns of gray level intensities in images, and were impossible to recognizable by the human eye [20,26]. Many articles have proven that MRI-derived texture features could predict placental diseases and depend less on experienced radiologists, especially those facing doubtful cases of PAS, which were better than conventional diagnostic imaging [15,16,17,18,26]. Hence, we developed prediction models based on radiomics to make intraoperative risk evaluation for guiding treatment that is superior to the previous evaluation based on imaging diagnosis. 

### 3.3. The Necessity of Combined Clinical Variable Analysis for Intraoperative Risk Assessment

Considering that prior CS and placenta previa are the most important clinical information for patients with PAS, we finally adopted a clinical-radiomics combined model to evaluate intraoperative risk. The AUC of the combined models was maximal. The prediction model for option of RPP included positive clinical variables of check-in gestational week and placenta previa. Placenta previa was highly correlated with PAS and became the variable of the model, which was understandable [27]. As an additional positive clinical variable, check-up gestational week was present in the model. We speculated that the check-up gestational week represented the time of suspected PAS in patients. This indicates that the earlier the PAS is suspected, the higher the probability of PAS in patients. In addition, placenta previa and the number of previous CS were adopted to construct the prediction model for IBL. The reason for this was that the region of previous CS is prone to forming hypervascularity in the follow-up pregnancy, which leads to an increase in the amount of bleeding [28].

### 3.4. The Clinical Significance of Intraoperative risk Assessment

The nomogram intuitively lists important clinical characteristics and the Rad score. For each patient, we can calculate the risk percentage based on the nomogram, as shown in Figure 2, to realize personalized treatments. Furthermore, the results of the DCA indicate that using the based-combined model nomogram could obtain greater net benefits than the clinical model alone at the threshold probabilities of 12–88% for the selection of RPP and a threshold probability of 2–69% as well as 74–92% for the prediction of IBL.

### 3.5. Limitation

This study has several limitations. First, the sample size was limited. An analysis with a larger sample size will be able to obtain more accurate models. External validation is also needed to confirm the performance of the prediction model. Second, we could not obtain all the pathological samples because some patients received CS as well as placental separation and not hysterectomy. Third, segmentation bias was unavoidable in the analysis images. In spite of this, certain measures to avoid potential selection bias in the analyses were undertaken. Finally, it was a retrospective analysis based on operative results of high-risk patients with previously performed MRI scans. Although, all the patients received CS delivery within a week after MRI examination, in theory, information of previous MRI does not truly reflect operative results. It is difficult to avoid in clinical practice.

## 4. Materials and Methods

### 4.1. Patients

**Figure 4:**
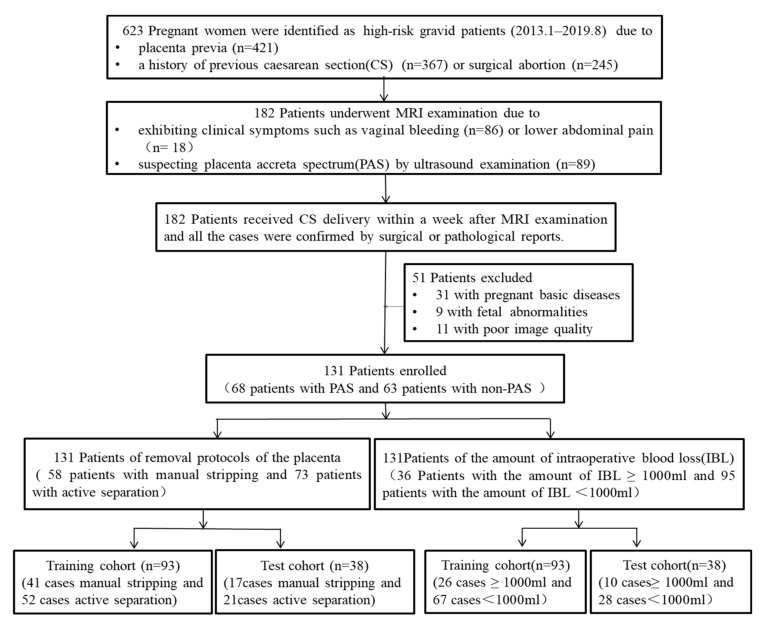
The workflow of this study.

This study was approved by our institutional ethics committee (XHEC-D-2021-143), and the requirement for informed consent was obtained. From January 2013 to August 2019, 623 high-risk gravid patients who had a history of prior CS or surgical abortion and present placenta previa, underwent regular prenatal examinations at our hospital. Among them, 182 patients underwent MRI examination due to exhibiting clinical symptoms including vaginal bleeding or lower abdominal pain or suspecting PAS by US. Finally, all the patients received CS delivery within a week after MRI examination and all the cases were confirmed by either surgical gross findings or pathological results. Fifty-one patients with pregnant basic diseases, fetal abnormalities, and poor image quality were excluded. Consequently, 131 patients were enrolled in the study, and 68 of them were diagnosed of PAS and 63 of non-PAS. Further, their data were retrospectively analyzed. All recruited patients were randomly assigned to the training and test cohorts at a ratio of 7:3. Subsequently, qualitative and quantitative images as well as clinical evaluations were performed. The workflow of this study is illustrated in Figure 4.

### 4.2. Assessment Standard of Intraoperative Risks

All the patients received just standard CS from the lower uterine segment approach. After synthetic oxytocin administration as well as gentle traction of the cord, the placenta of some patients can be actively detached, which was defined as active separation of the placenta. In turn, the placenta of the others required to be manually removed, which was viewed as manual stripping of the placenta. In addition, only few patients further received hysterectomy owing to serious conditions. In the study, intraoperative risk was defined as the manual stripping of the placenta and accompanying major bleeding during the caesarean procedure, excluding hysterectomy or postpartum hemorrhage. Assessment standard from two aspects was established according to operation records. The first one was selection criteria of removal protocols of the placenta (RPP), which were classified as active separation and manual removal of the placenta, depending on the depth of placental invasion. The second one was prediction criteria of intraoperative blood loss (IBL), in which, 1000 mL was considered the grouped criterion for amount of IBL. PAS diagnostic reference included the intraoperative gross findings (surgical evidence) and the histopathological findings.

### 4.3. MRI Imaging

All the patients underwent pelvic MRI using a 1.5-T scanner (GE Medical System Milwaukee, Wisconsin, USA) with a Torso coil in the supine position. The imaging sequences included sagittal and coronal FIES-TA, and axial fast inversion recovery motion insensitive (FIRM) and FIESTA. Their detailed scan parameters were shown below. Flip angle and echo time (TE)/repetition time (TR) of FIESTA and FIRM were respectively 60° versus 55°; 1.6–1.8 ms/3.6–3.9 ms and 2.0–5.3 ms/7.7–10.7 ms; thickness and slice interval were 4–5 mm and 0–2 mm, with a matrix of 224 × 224 and a field of view of 360–420 mm.

### 4.4. Radiomics Analysis

#### 4.4.1. Image Analysis and Segment

**Figure 5:**
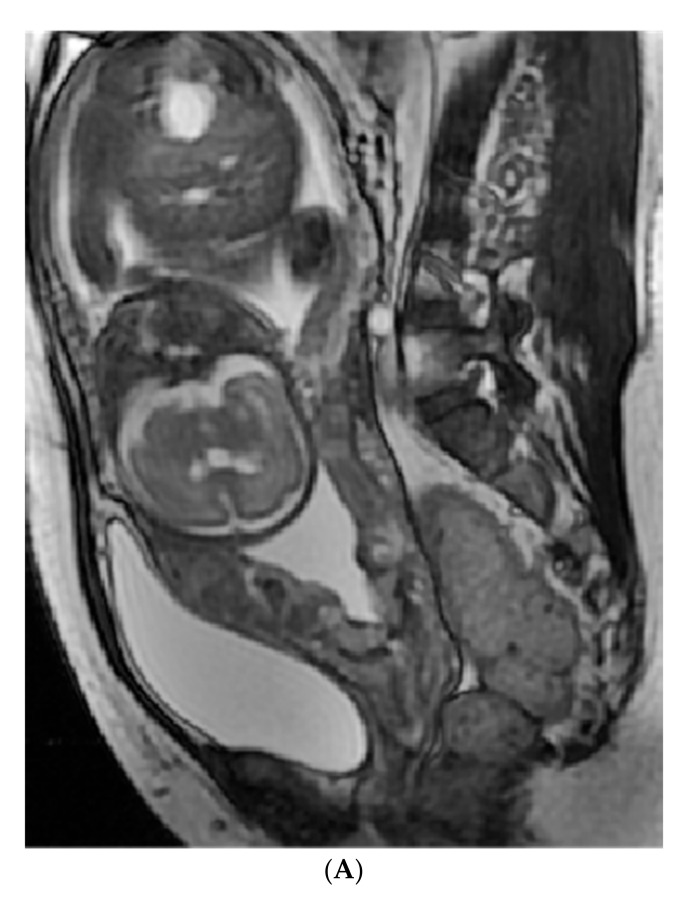

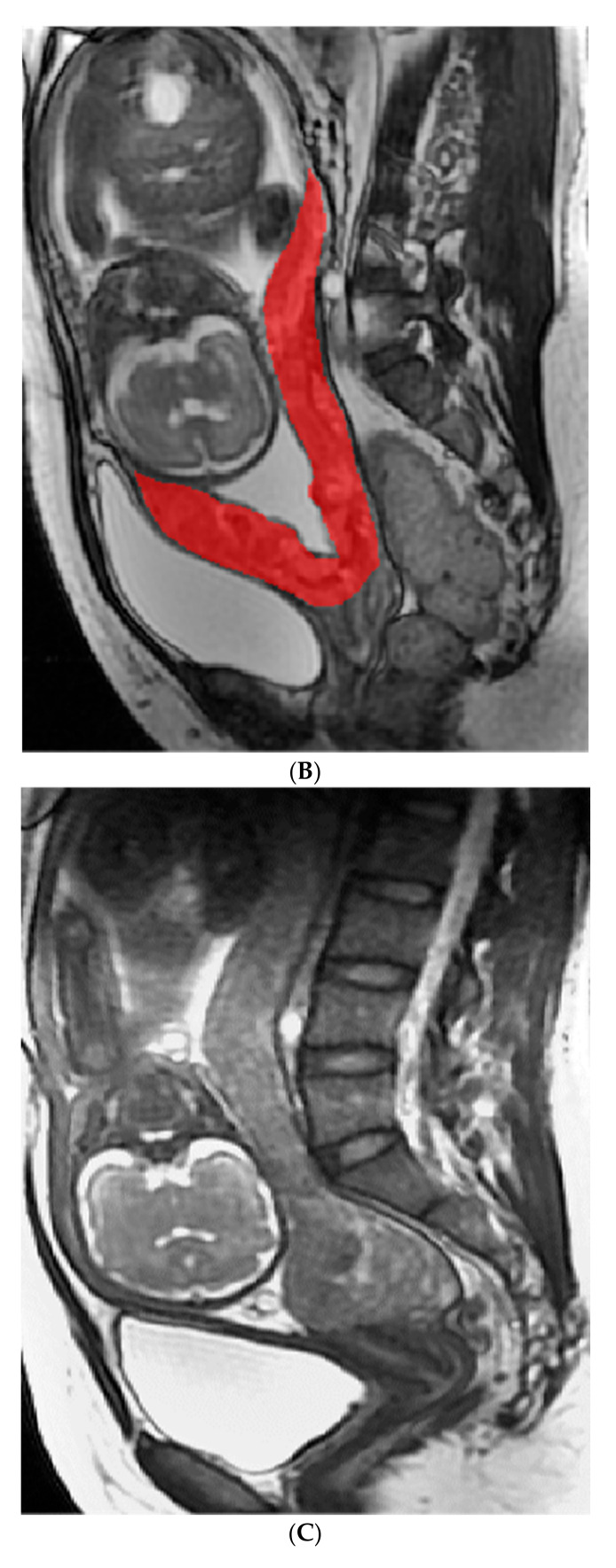

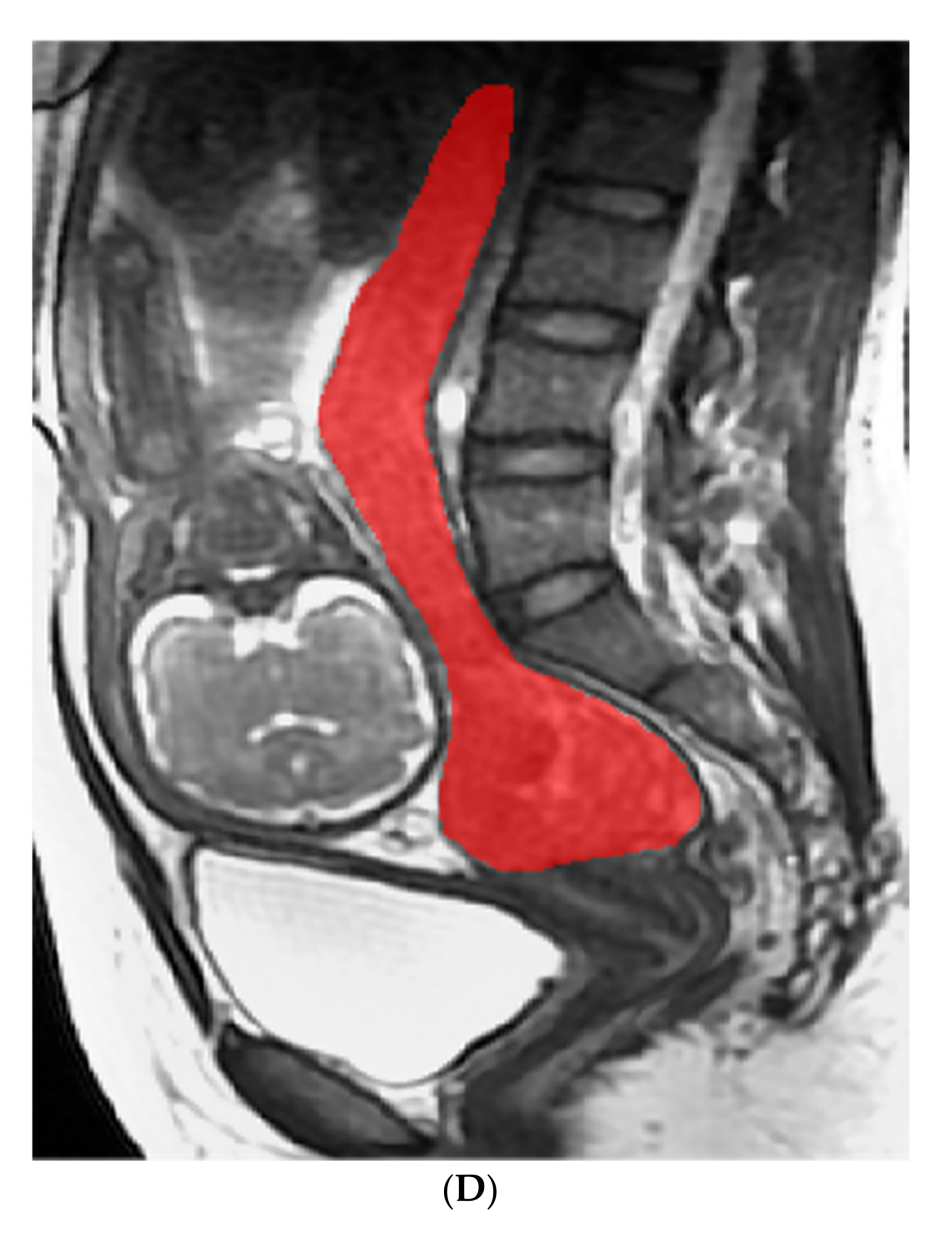
(**A**,**B**): A 36-year-old patient with placenta increta, receiving manual stripping of the placenta along with the amount of IBL of up to 5000 mL. The red region shows the VOI of delineation including the placenta and uterus; Weeks of gestation at time of MRI examination: 35.4/10 points; Placenta previa: Complete/42 points; Rad score: 0.64/58 points; Total score: 110 points; The risk of manual stripping placenta: 0.9; Number of CS: 1/48 points; Placenta previa: Complete/88 points; Rad score: 3.2/100 points; Total score: 236 points; The risk of the amount of IBL more than 1000 mL: >0.9; (**C**,**D**): A 23-year-old patient with placenta accreta, who underwent active separation of the placenta along with the amount of IBL of 350 mL. The red region shows the VOI of delineation including the placenta and uterus; Weeks of gestation at time of MRI examination: 30.9/20 points; Placenta previa: Partial/28 points; Rad score: −2.4/0 points; Total score: 48 points; The risk of manual stripping placenta: 0.2; Number of CS: 0/0 points; Placenta previa: Partial/60 points; Rad score: −0.76/10 points; Total score: 70 points; The risk of the amount of IBL more than 1000 mL: 0.15.

The sagittal FIESTA sequence as the optimal position for pelvic observation was applied for MRI image analysis and segmentation. For consistency, two board-certified obstetric radiologists (with 7 and 8 years of experience), who were blinded to the clinic-pathological information, together analyzed the sagittal images of the patients, and identified the regions and the maximum margin of suspicious lesions from the placenta and uterus by reaching a consensus. Then, the volume of interest (VOI) was manually delineated by the third radiologists (with 12 years of experience) with reference to the previous consensus and via a free open-source software package (itk-SNAP, version 3.4.0, www.itksnap.org, accessed on 20 November 2020,), as shown in Figure 5.

#### 4.4.2. Radiomics Feature Extraction, Selection, and Radiomics Score Calculation

A total of 1130 radiomic features from the VOI were extracted automatically using an in-house software (Analysis Kit, version 3.0.0, GE Healthcare, Shanghai, China). Two feature selection methods, the minimum redundancy maximum relevance (mRMR) algorithm and the least absolute shrinkage and selection operator (LASSO) regression, were used [16,25]. The mRMR was performed to eliminate redundant and irrelevant features, and LASSO was conducted to choose an optimized subset of features. Considering the imbalance of the data, we used SMOTE for the oversampling of the small number parts; critical radiomic features were subsequently obtained. The radiomics score (Rad score) was calculated for each high-risk patient using a linear combination of selected radiomic features and their weighted coefficients. 

### 4.5. Development of the Radiomics, Clinical, and Clinical-Radiomics Combined Models

The radiomics and clinical models were built by univariate and multivariate logistic regression analyses based on the obtained critical radiomic features and clinical factors in the training cohort. The clinical-radiomics combined model and clinical-radiomics nomogram were constructed with the selected clinical variables and Rad score in the training cohort. Subsequently, all the models were validated using the test cohort.

### 4.6. Evaluating the Performance and Utility of All the Models

Receiver operating characteristic curve (ROC) analysis was used to evaluate the predictive performance of the radiomics, clinical, and combined models. The area under the curve (AUC), accuracy, sensitivity, specificity, positive predictive value (PPV), and negative predictive value (NPV) were calculated. The predictive performance of the models was validated in the test cohort. Decision curve analysis (DCA) was employed to assess the net benefits of all models for different threshold probabilities in the entire cohort.

### 4.7. Statistical Analysis

For continuous variables, Kolmogorov–Smirnov test was firstly used to analyze normal distribution. Then the continuous variables were analyzed using the t-test and categorical variables using Chi-squared test in the two groups of removal protocols of the placenta and in the two groups of the amount of bleeding during the operative course, by SPSS 23.0. Python was used to extract and select the radiomic features. Univariate and multivariate regression analyses for prediction model building were performed using R software (Shanghai, China, http://www.Rproject.org accessed on 15 March 2021). ROC analysis was performed to determine the performance of the models via the “pROC” package and all the evaluation indicators such as accuracy, sensitivity, specificity, and so on were calculated based on the Youden Index. Decision curve analysis was performed using the “rmda” package. Statistical significance was set at *p* < 0.05.

## 5. Conclusions

In conclusion, our study showed that prediction models based on radiomics as well as clinical variables can accurately predict RPP and IBL for gravid patients at high risk with PAS. The clinical-radiomics nomogram as an individual and visualized tool could offer precise and personalized management protocols for patients.

## Figures and Tables

**Table 1:** Characteristics of patients and their associations with removal protocols of the placenta.

Characteristics	Training Cohort (n = 93)			Testing Cohort (n = 38)		
	Active Separation (N = 52)	Manual Stripping (N = 41)	*p*-Value	Active Separation (N = 21)	Manual Stripping (N = 17)	*p*-Value
**Age** (mean ± SD)	31.90 ± 4.53	32.76 ± 4.56	0.37	32.10 ± 4.33	33 ± 3.76	0.16
**Weeks of gestation at time of MRI examination **(mean ± SD)	34.54 ± 3.03	30.74 ± 7.37	<0.001	34.86 ± 2.60	33.35 ± 4.45	0.20
**Vaginal bleeding n (%)**			0.27			0.04
No	34 (65.4)	20 (48.8)		13 (61.9)	6 (35.3)	
Minor	16 (30.8)	19 (46.3)		6 (28.6)	11 (64.7)	
Massive	2 (3.8)	2 (4.9)		2 (9.5)	0 (0)	
**Placenta previa n (%)**			<0.001			0.20
Low lying	20 (38.5)	31 (75.6)		8 (38.2)	12 (70.6)	
Marginal	11 (21.2)	3 (7.3)		4 (19)	2 (11.8)	
Partial	10 (19.2)	3 (7.3)		4 (19)	2 (11.8)	
Complete	11 (21.2)	4 (9.8)		5 (23.8)	1 (5.9)	
**No. of previous CS n (%)**			0.01			<0.001
=0	36 (69.2)	18 (43.9)		1 (85.7)	6 (35.3)	
=1	16 (30.8)	19 (46.3)		3 (14.3)	10 (58.8)	
≥2	0 (0)	4 (10)		0 (0)	1 (5.9)	
**No. of previous surgical abortion n (%)**			0.01			0.31
=0	33 (63.5)	13 (31.7)		10 (47.6)	6 (35.3)	
=1	11 (21.2)	19 (46.3)		7 (33.3)	10 (58.8)	
≥2	8 (15.3)	9 (22)		4 (19.1)	1 (5.9)	
**Blood loss during surgery** (mean ± SD mL)	471.54 ± 296.74	1834.88 ± 1774.03	<0.001	379.52 ± 96.67	2047.06 ± 2041.18	<0.001
**PAS**			<0.001			<0.001
No	37 (71.2)	3 (7.3)		20 (95.2)	3 (17.6)	
Placenta accrete	15 (28.8)	10 (24.4)		1 (4.8)	5 (29.4)	
Placenta increta	0 (0)	24 (58.5)		0 (0)	7 (41)	
Placenta percreta	0 (0)	4 (9.8)		0 (0)	2 (12)	

CS, cesarean section; PAS, placenta accreta disorder.

**Table 2:** Characteristics of patients and their associations with intraoperative blood loss.

Characteristics	Training Cohort (n = 93)			Testing Cohort (n = 38)		
	<1000 mL (N = 67)	≥1000 mL (N = 26)	*p*-Value	<1000 mL (N = 28)	≥1000 mL (N = 10)	*p*-Value
**Age **(mean ± SD)	31.63 ± 4.47	32.85 ± 4.30	0.34	32.86 ± 4.64	32.3 ± 3.74	0.74
**Weeks of gestation at time of MRI examination (mean ± SD)**	34.24 ± 3.80	29.88 ± 7.48	<0.001	34 ± 4.38	33.25 ± 5.25	0.66
**Vaginal bleeding n (%)**			0.67			0.71
No	42 (62.7)	10 (38.5)		15 (53.6)	6 (60)	
Minor	22 (32.8)	14 (53.8)		12 (42.9)	4 (40)	
Massive	3 (4.5)	2 (7.7)		1 (3.6)	0 (0)	
**Placenta previa n (%)**			<0.001			0.04
Low lying	26 (38.8)	24 (92.3)		13 (46.4)	8 (80)	
Marginal	14 (20.9)	0 (0)		6 (21.4)	0 (0)	
Partial	12 (17.9)	1 (3.8)		4 (14.3)	2 (20)	
Complete	15 (22.4)	1 (3.8)		5 (17.9)	0 (0)	
**No. of previous CS n (%)**			<0.001			0.01
=0	49 (73.1)	7 (26.9)		20 (71.4)	2 (20)	
=1	16 (23.9)	17 (65.4)		8 (28.6)	7 (70)	
≥2	2 (3.0)	2 (7.7)		0 (0)	1 (10)	
**No. of previous surgical abortion n (%)**			0.02			0.09
=0	39 (58.2)	7 (27.0)		13 (46.4)	3 (30)	
=1	17 (25.4)	14 (53.8)		9 (32.1)	7 (70)	
≥2	11 (16.4)	5 (19.2)		6 (21.5)	0 (0)	
**Removal protocols of the placenta**			<0.001			<0.001
Active separation	48 (71.6)	3 (11.5)		21 (75)	1 (10)	
Manual stripping	19 (28.4)	23 (88.5)		7 (25)	9 (90)	
**PAS**			<0.001			<0.001
No	48 (71.6)	1 (3.8)		14 (50)	0 (0)	
Placenta accrete	14 (20.9)	2 (7.7)		12 (42.9)	3 (30)	
Placenta increta	5 (7.5)	18 (69.2)		2 (7.1)	6 (60)	
Placenta percreta	0 (0)	5 (19.2)		0 (0)	1 (10)	

CS, cesarean section; PAS, placenta accreta disorder.

**Table 3:** Performances of all the predictive models.

Evaluation-Parameters	The Predictive Model for Removal Protocols of The Placenta		The Predictive Model for Intraoperative Blood Loss	
	Training Cohort	Test Cohort	Training Cohort	Test Cohort
**Radiomics model**				
AUC (95%CI)	0.87 (0.79~0.94)	0.86 (0.74~0.98)	0.86 (0.78~0.94)	0.86 (0.73~1.0)
Accuracy (95%CI)	0.85 (0.76~0.92)	0.79 (0.63~0.90)	0.83 (0.74~0.90)	0.87 (0.72~0.96)
Sensitivity	0.90	0.76	0.85	0.93
Specificity	0.78	0.82	0.77	0.70
Pos. Pred. Value	0.84	0.84	0.90	0.90
Neg. Pred. Value	0.86	0.74	0.67	0.78
**Clinical model**				
AUC (95%CI)	0.72 (0.61~0.83)	0.69 (0.51~0.86)	0.84 (0.76~0.92)	0.79 (0.65~0.94)
Accuracy (95%CI)	071 (0.54~0.85)	0.69 (0.58~0.78)	0.76 (0.60~0.89)	0.82 (0.72~0.89)
Sensitivity	0.71	0.63	0.55	0.68
Specificity	0.71	0.74	0.85	0.87
Pos. Pred. Value	0.59	0.71	0.6	0.65
Neg. Pred. Value	0.81	0.67	0.82	0.88
**Combined model with radiomics and clinics**				
AUC (95%CI)	0.90 (0.83~0.97)	0.91 (0.82~1.00)	0.90 (0.84~0.96)	0.88 (0.77~0.99)
Accuracy (95%CI)	0.87 (0.79~0.93)	0.87 (0.72~0.96)	0.81 (0.77~0.88)	0.76 (0.60~0.89)
Sensitivity	0.94	1.0	0.6	0.53
Specificity	0.83	0.81	0.96	0.95
Pos. Pred. Value	0.76	0.71	0.92	0.9
Neg. Pred. Value	0.96	1.0	0.76	0.71

AUC, area under the receiver operating characteristic curve; CI, confidence interval.

## Data Availability

The datasets used and/or analyzed during the current study are available from the corresponding author on the reasonable request.

## Supplementary Materials

The following are available online at https://www.mdpi.com/article/10.3390/diagnostics12020485/s1, Figure S1: title, Selection of Radiomics features using the least absolute shrinkage and selection (LASSO) regression for predicting removal protocols of the placenta (A) and intraoperative blood loss (B).
