# Supplementary material for: MRI-Based Radiomics Analysis for Intraoperative Risk Assessment in Gravid Patients at High Risk with Placenta Accreta Spectrum
Source: Diagnostics (Basel). 2022 Feb 14;12(2):485. doi: 10.3390/diagnostics12020485 (PMC8870740; doi:10.3390/diagnostics12020485)
Supplement: Supplementary file 1 [file diagnostics-12-00485-s001.zip › Supplementary Figure S1 caption.pdf]

Supplementary Figure S1: Selection of Radiomics features using the least absolute shrinkage and selection (LASSO) regression for predicting removal protocols of the placenta (A) and intraoperative blood loss (B).
